# Editorial: Third International Tenebrionoidea Symposium

**DOI:** 10.3897/zookeys.415.7113

**Published:** 2014-06-12

**Authors:** Aaron D. Smith, Rolf L. Aalbu, Patrice Bouchard

**Affiliations:** 1 Department of Biological Sciences, Northern Arizona University, PO Box 5640, Flagstaff, AZ, 86011-5640, USA; 2 Department of Entomology, California Academy of Sciences, 55 Music Concourse, Dr., Golden Gate Park, San Francisco, California, U.S.A.; 3 Canadian National Collection of Insects, Arachnids and Nematodes, Agriculture and Agri-Food Canada, 960 Carling Avenue, Ottawa, Ontario, K1A 0C6, Canada

The Third International Tenebrionoidea Symposium (ITS) was held at Arizona State University in Tempe, Arizona USA on August 7th and 8th, 2013. Researchers from ten countries participated with a total of 36 attendees (Figure 1). It was the first formal meeting of the international tenebrionoid research community since the October 2005 in Lyon, France. Though the previous meetings did not list themselves as the beginning of a series, we consider it fitting to acknowledge them as the first two modern international meetings specifically organized for the sharing and dissemination of Tenebrionoidea research.

**Figure 1.**
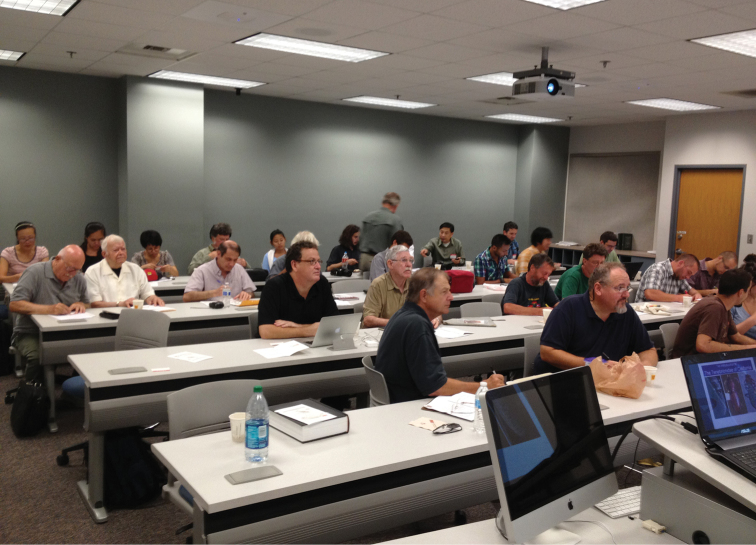
August 7th, before the first talk.

The 1st International Tenebrionid Symposium, entitled “Systematics and Biogeography of Tenebrionoidea”, was held in 2002 at the Zoologisches Staatssammlung, München (Germany) to honor Dr. Hans J. Bremer’s work on tenebrionids and celebrate the museum’s acquisition of his collection. This event organized by Dr. Martin Baehr resulted in a highly successful meeting.

The 2nd International Tenebrionoidea Symposium, entitled “Coleoptera Tenebrionoidea: Taxonomy, Biogeography, and Faunistics”, was held in 2005 at the Lyon Museum (France) following the acquisition of the remarkable tenebrionid collection of Jaroslav Picka. Following the symposium, many of the presentations were published in Cahiers Scientifiques (Fascicule 10). Again a highly successful meeting this time organized by Dr. Harold LaBrique.

To continue this successful tradition, and encourage tenebrionoid workers from around the world to meet, share their research, and form new collaborations, researchers in the US and Canada decided to host the 3nd International Tenebrionoidea Symposium. A steering committee was assembled with representatives from Arizona State University, California Academy of Sciences, the Canadian National Collection of Insects, and the Smithsonian Institution. Arizona State University in Tempe, Arizona was ultimately chosen to host the symposium due to its institutional support, excellent facilities, and multiple opportunities for field work both before and after the meeting. Presentations were given on August 7th and 8th, 2013.

Before the meeting, researchers visited US collections on both the west and east coasts and held a pre-meeting collecting trip through California, Nevada, Utah, and Arizona. Gustavo Flores had the most impressive itinerary of museum visits. After flying into New York City from Mendoza, Argentina, Gustavo visited the American Museum of Natural History (AMNH – New York, New York), the Smithsonian Institution (NMNH – Washington, D.C.), the C.A. Triplehorn Insect Collection at Ohio State University (OSUC – Columbus, Ohio), the Field Museum (FMNH – Chicago, Illinois), and Rolf Aalbu’s personal collection (RLAC – El Dorado Hills, California). In Sacramento, Gustavo joined Wolfgang Schawaller, Roland Grimm, and René Fouquè who had been working in the California Academy of Sciences (CASC – San Francisco, California), California Department of Food and Agriculture (CDFA – Sacramento, California), and RLAC collections the prior week. Rolf, Gustavo, René, Roland, and Wolfgang then drove from Sacramento to Tempe while doing field work through California, Nevada, Utah, and north central Arizona (Figure 2).

**Figure 2.**
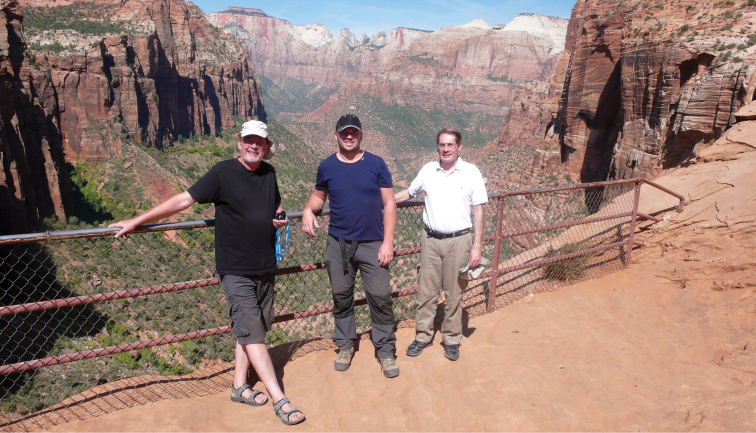
Pre-meeting sightseeing stop at Zion National Park, Utah. Left to right: Wolfgang Schawaller, René Fouquè, Gustavo Flores.

During the meeting 21 presentations, seventeen 20-minute talks and four posters, where given (see http://insectbiodiversitylab.org/3ITS_presentations.html) ranging from species-level revisions to broad scale Tenebrionidae phylogenies and inventories, darkling beetles intercepted by USDA-APHIS during agricultural quarantine interceptions, and the first steps towards the construction of a Coleopteran Anatomy Ontology. Presentations were generally well received and elicited animated question and answer sessions.

Many of the attendees had previously corresponded by email, but never met in person. For example, Guodong Ren’s research group (Figure 3) has been remarkably productive, but this was the first time any of the American (North and South) or European visitors were able to meet him face to face. Others, such as Chuck Triplehorn (Figure 4) are well known to almost all attendees through both research and previous visits. Following the first day’s presentations, Bill Warner led an evening collecting expedition to Oak Flat Campground in Pinal County.

**Figure 3.**
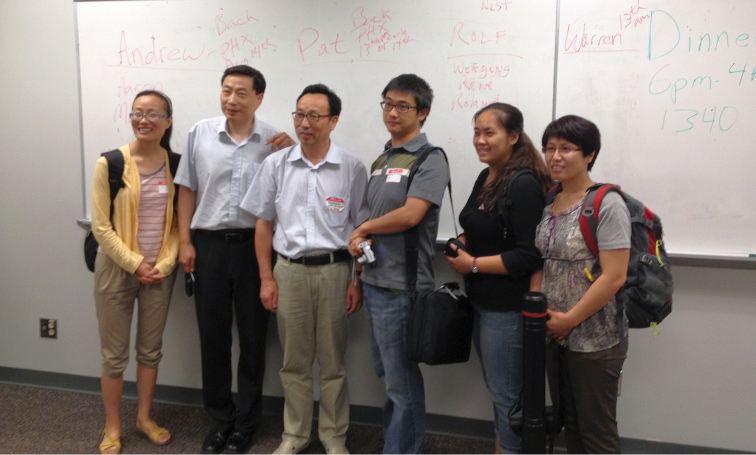
Visiting Chinese and US-based Chinese researchers. Left to right: Yuxia Yang, Li Zhong, Guodong Ren, Guanyang Zhang (ASU postdoc), Shanshan Liu, Caixia Yuan

**Figure 4.**
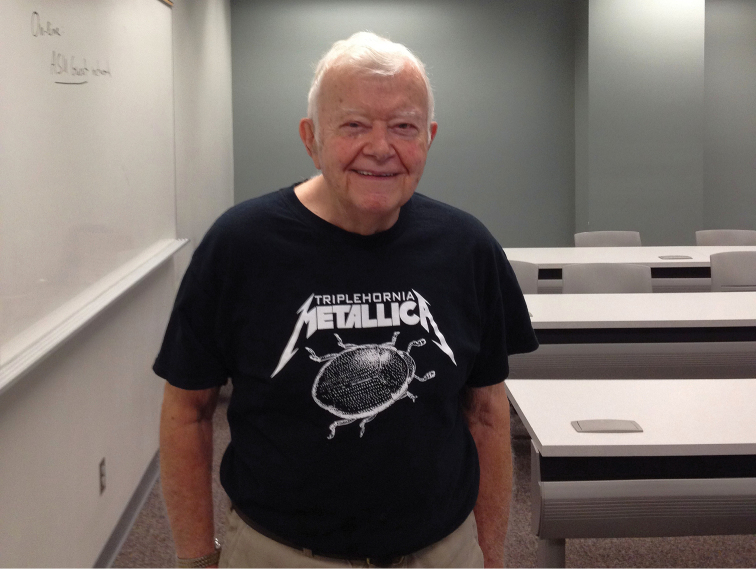
Dr. Charles A. Triplehorn showing off a Triplehornia metallica Matthews and Lawrence shirt made by his grandson.

Group discussions were also held during the symposium on potential large scale tenebrionid projects that could be undertaken as a community, the organization of a Proceedings volume from the Symposium, collecting localities for the post-meeting trip, and potential localities and dates for the Fourth International Tenebrionoidea Symposium. Informal talks on these and other tenebrionoid related matters extend far into the evening and past the closing session on August 8th (Figure 5). Pat Bouchard agreed to act as lead editor for a Proceedings volume in the journal Zookeys, for which we were and remain very grateful. Most articles included in this resulting special issue of ZooKeys are based on the contents of presentations during the Third International Tenebrionoidea Symposium, although papers submitted by all attendants were also welcome.

**Figure 5.**
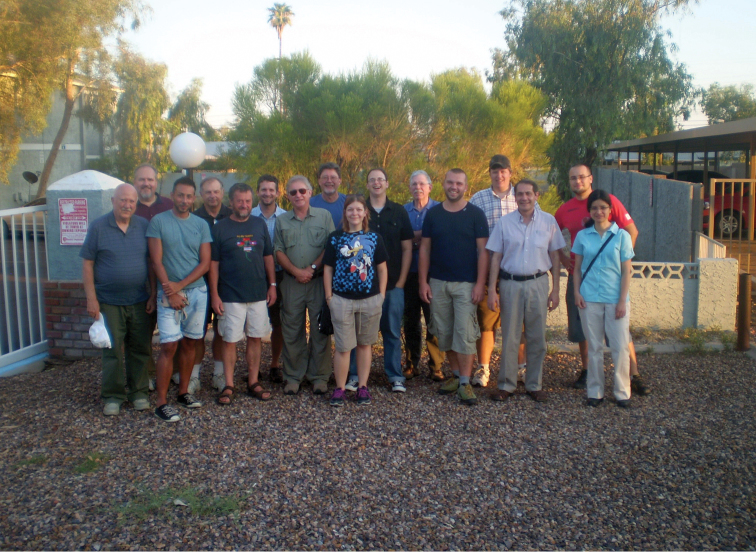
Post meeting dinner. Left to right, back row: Bill Warner, Rich Cunningham, Pat Bouchard, Wolfgang Schawaller, Aaron Smith, Milton Campbell, Andrew Johnston, Marcin Kamiński; front row: Ron Somerby, Gael Kergoat, Roland Grimm, Rolf Aalbu, Rebecca Dornburg, René Fouquè, Gustavo Flores, Paulina Cifuentes Ruiz.

After the formal symposium, attendees went their separate ways, with some doing solo collecting and some visiting US museums (California Academy of Sciences and the University of Arizona Insect Collection to name just two). Twelve researchers from five countries went to the Beetle Infestation VI on August 10th hosted by Pat and Lisa Sullivan in Ramsey Canyon, Huachuca Mountains, one of the most biologically diverse localities in the United States, before collecting through southern and central Arizona (Figures 6 & 7) eventually disbursing into smaller field groups or heading home. While a full tally of tenebrionoid species collected in association with the symposium will likely never be assembled, the first author collected approximately 40 darkling beetles species during and after the meeting. Most of the species collected can be sight IDed, at least to genus, using Bugguide.

**Figure 6.**
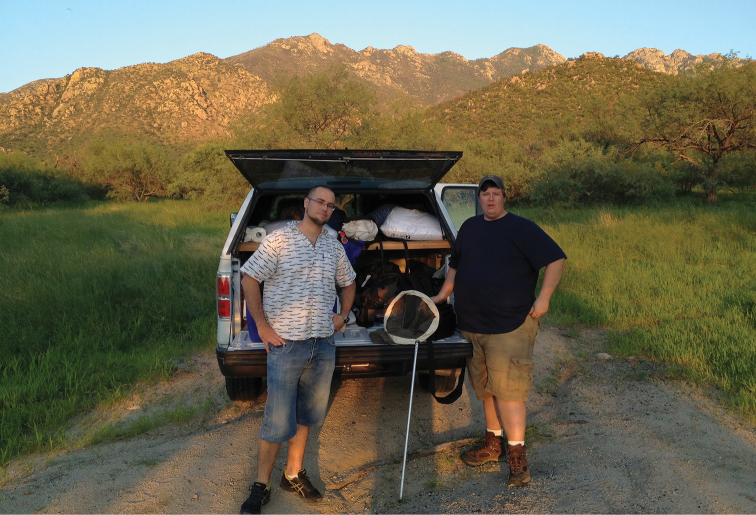
Post meeting collecting. Marcin Kamiński and Andrew Johnston near Madera Canyon.

**Figure 7.**
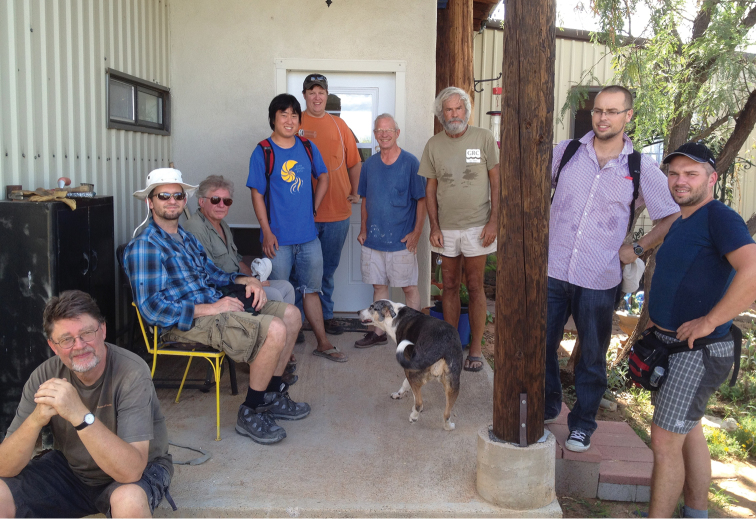
Post meeting afternoon break at Fred Skillman’s house, Cochise, AZ. Left to right: Wolfgang Schawaller, Pat Bouchard, Rolf Aalbu, Kojun Kanda, Andrew Johnston, Fred Skillman, Warren Steiner, Marcin Kamiński, René Fouquè.

Many of the presentations, a list of collecting localities, and additional pictures from the symposium are online at: http://www.insectbiodiversitylab.org/3ITS.html.

Two researchers graciously volunteered to host the next symposium at their institutions: Gustavo Flores (CCT CONICET – Mendoza, Argentina) for 2016, or Guodong Ren (Hebei University – Baoding City, China) for 2015, and presented short talks highlighting the advantages of their respective cities. A survey was set up to allow the attendees of the Third symposium, current tenebrionoid researchers (those with at least one tenebrionioid manuscript in print), and graduate students working on tenebrionoids to vote for the host city of the Fourth International Tenebrionoidea Symposium. Voting was open until September 30th, 2013 and turnout was excellent. After over a month of voting, Mendoza, Argentina was chosen to host the next meeting in November 2015. See you in Mendoza!

## Steering committee

Rolf Aalbu – California Academy of Science, San Francisco, CA USA

Patrice Bouchard – Canadian National Collection of Insects, Ottawa, Ontario CANADA

Kojun Kanda – Oregon State University, Corvallis, OR USA

Nico Franz – Arizona State University, Tempe, AZ USA

Aaron Smith – Arizona State University, Tempe, AZ USA

Warren Steiner – Smithsonian Institution, Washington, DC USA

Quentin Wheeler – Arizona State University, Tempe, AZ USA

## Attendees (Current country of residence)

Rolf L. Aalbu (USA)

Jason T. Botz (USA)

Patrice Bouchard (Canada)

Milton Campbell (Canada)

Alejandro Castro Tovar (Spain)

Paulina Cifuentes Ruiz (Mexico)

Adriana Li Colinas (Spain)

Rich Cunningham (USA)

Rebecca Dornburg (USA)

Gustavo E. Flores (Argentina)

Rene Fouque (Czech Republic)

Nico M. Franz (USA)

Roland Grimm (Germany)

Andrew Jansen (USA)

Andrew Johnston (USA)

Marcin Jan Kamiński (Poland)

Kojun Kanda (USA)

Gael J. Kergoat (France)

Shanshan Liu (China)

James Pflug (USA)

Guodong Ren (China)

Wolfgang Schawaller (Germany)

Aaron D. Smith (USA)

Ronald Somerby (USA)

Warren Steiner (USA)

Larry Stevens (USA)

Donald Thomas (USA)

Donald Thomas, Jr. (USA)

Charles A. Triplehorn (USA)

Bill Warner (USA)

Quentin Wheeler (USA)

Yuxia Yang (China)

Caixia Yuan (China)

Guanyang Zhang (USA)

Patricia Eri Ishii (USA)

